# Effects of antibiotic suppression on three healthcare systems’ National Healthcare Safety Network Antibiotic Resistance Option data

**DOI:** 10.1017/ash.2021.202

**Published:** 2021-11-10

**Authors:** Christopher D. Evans, Matthew D. Estes, Youssoufou Ouedraogo, Daniel Muleta, Marion A. Kainer, Pamela P. Talley

**Affiliations:** 1 Communicable and Environmental Diseases and Emergency Preparedness Division, Healthcare-Associated Infections and Antimicrobial Resistance Program, Tennessee Department of Health, Nashville, Tennessee, United States; 2 Department of Infectious Diseases, Western Health, Footscray, Victoria, Australia

## Abstract

**Objective:**

The National Healthcare Safety Network (NHSN) Antibiotic Resistance (AR) Option is a valuable tool that can be used by acute-care hospitals to track and report antibiotic resistance rate data. Selective and cascading reporting results in suppressed antibiotic susceptibility results and has the potential to adversely affect what data are submitted into the NHSN AR Option. We describe the effects of antibiotic suppression on NHSN AR Option data.

**Methods:**

NHSN AR Option data were collected from 14 hospitals reporting into an existing NHSN user group from January 1, 2017, to December 31, 2018, and linked to commercial automated antimicrobial susceptibility testing instruments (cASTI) that were submitted as part of unrelated Tennessee Emerging Infections Program surveillance projects. A susceptibility result was defined as suppressed if the result was not found in the NHSN AR Option data but was reported in the cASTI data. Susceptibility results found in both data sets were described as released. Proportions of suppressed and released results were compared using the Pearson χ^2^ and Fisher exact tests.

**Results:**

In total, 852 matched isolates with 3,859 unique susceptibilities were available for analysis. At least 1 suppressed antibiotic susceptibility result was available for 726 (85.2%) of the isolates. Of the 3,859 susceptibility results, 1,936 (50.2%) suppressed antibiotic susceptibility results were not reported into the NHSN AR option when compared to the cASTI data.

**Conclusion:**

The effect of antibiotic suppression described in this article has significant implications for the ability of the NHSN AR Option to accurately reflect antibiotic resistance rates.

The Centers for Disease Control and Prevention (CDC) estimates that >2.8 million antibiotic-resistant infections occur each year, resulting in >35,000 deaths.^1^ A main objective of the US Government National Action Plan for Combating Antibiotic-Resistant Bacteria (CARB) is to expand the availability of antibiotic resistance surveillance data.^2^ Acute-care hospitals routinely track antibiotic resistance data, often reporting these data institution-wide in the form of an antibiogram. Significant work on the part of the institution’s microbiology laboratory is required to compile, clean, analyze, and present antibiotic resistance data. The National Healthcare Safety Network (NHSN) AR Option provides a means for acute-care hospitals to compile and report antibiogram-level data. Eligible susceptibility reports are uploaded electronically via each facility’s laboratory information management system (LIMS) or electronic medical record (EMR), often via a commercial software surveillance system. This method is promoted in the 2020 CARB report.^2^ State and local health departments can also leverage the NHSN AR Option data to track antibiotic resistance at the state and regional level.^3^ The Tennessee Department of Health (TDH) established access to statewide acute-care facility NHSN AR data through their NHSN user group. By December 31, 2018 (the end of the study period), 22 acute-care hospitals across 5 regions in the state had conferred data access rights to the TDH.

Two examples of microbiology-based interventions that acute-care stewardship teams may adopt to drive appropriate antibiotic use include selective and cascading reporting of antibiotic susceptibility results.^4^ With selective reporting and working with microbiology laboratory leadership, stewardship programs decide which selected antibiotics to show on the laboratory report. With cascading reporting, antibiotics shown are based on the other susceptibility results of the organism. Both methods encourage prudent prescribing and discourage the use of nonpreferred agents. The decision regarding which antibiotics to include or suppress is made by members of the antibiotic stewardship, infectious diseases, and microbiology laboratory teams and is usually based upon antibiotic resistance patterns of the isolate in question, available formulary options, and hospital treatment guidelines. However, an unintended consequence of antibiotic susceptibility suppression may be incomplete reporting into the NHSN AR Option. Such suppressed reporting may limit the value of an NHSN AR Option–derived facility- or health-system–level antibiogram and the representativeness of the data for national antibiotic resistance surveillance purposes. We compared NHSN AR Option data with laboratory instrument data to determine the impact of antibiotic suppression of select agents on NHSN AR Option data.

## Methods

The Tennessee Department of Health (TDH) conducted a retrospective cross-sectional study using antibiotic susceptibility data collected from January 1, 2017, to December 31, 2018. We collected NHSN AR Option event data of facilities who voluntarily shared antibiotic resistance data through their pre-existing NHSN user group and from commercial automated antimicrobial susceptibility testing instruments (cASTIs) that were submitted as part of unrelated surveillance projects in the Tennessee Emerging Infections Program (EIP). The data from cASTIs were collected by querying the instrument before suppression occurred.

The cASTI data were matched to the NHSN AR Option data on 4 variables: microorganism, specimen date of collection, specimen source, and patient date of birth. Duplicate isolates, defined as an isolate that had the same 4 linkage variables as another observation, were removed. The following microorganisms were included in the analysis: carbapenem-resistant Enterobacterales (CRE) (including *Klebsiella* spp, *Enterobacter* spp, and *Escherichia coli* that were resistant to any tested carbapenem, based on 2017 Clinical and Laboratory Standards (CLSI) break points), extended-spectrum β-lactamase (ESBL)–producing Enterobacterales (including *Klebsiella* spp, and *Escherichia coli* that were resistant to at least 1 extended-spectrum cephalosporin, ie, ceftazidime, cefotaxime, or ceftriaxone, based on 2017 CLSI break points), *Pseudomonas aeruginosa*, and *Acinetobacter baumannii*.

We included the susceptibility results for carbapenems (imipenem-cilastatin, meropenem, and ertapenem) and third-generation cephalosporins (ceftriaxone, ceftazidime, and cefotaxime), which are most clinically relevant to these organisms. Ceftriaxone and cefotaxime results are not reportable to the NHSN AR Option for *Pseudomonas aeruginosa*, and these results were removed before analysis. The final susceptibility interpretation reported in both data sources was used to classify the isolates into 3 categories for each drug: susceptible, intermediate, and resistant. Isolates obtained from urine, blood, and respiratory specimens were included.

A susceptibility result was defined as suppressed if the result was not found in the NHSN AR Option data but was reported in the cASTI data. Alternatively, a susceptibility result found in both data sets was described as released. Isolates with no result in either data sets or isolates found in the NHSN AR Option data but not in the cASTI were classified as nonapplicable and were excluded from the analysis.

The proportion of suppressed data was calculated by dividing the number of isolates with suppressed results by the number of total isolates. The proportion of released data was calculated by dividing the number of isolates with released reports by the total number of isolates. These metrics were stratified by the healthcare system, the microorganism types, the most clinically relevant antibiotics, and the susceptibility pattern. The Pearson χ^2^ and Fisher exact tests were used to compare the proportions of suppressed and released data among the stratified classification variables. The statistical significance level was set at 0.05. All statistical analyses were performed using SAS version 9.4 software (SAS Institute, Cary, NC).

## Results

**Fig. 1.**
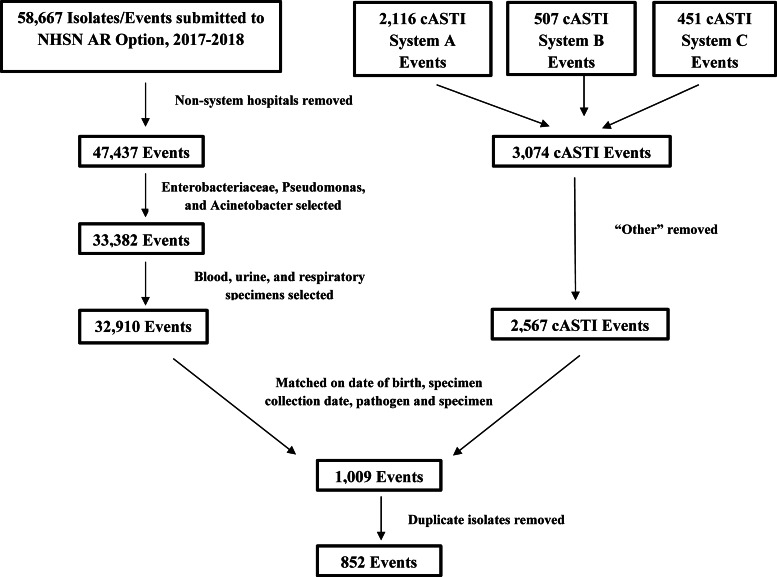
Data matching.

In total, 14 facilities from 3 healthcare systems that submitted data into the NHSN AR Option and reported cASTI data to TDH for EIP surveillance projects were included in this study. These facilities submitted susceptibility data for 47,437 isolates into the NHSN AR Option during 2017 and 2018 (Fig. 1). Also, 32,910 (69%) of these isolates were organisms targeted in EIP surveillance projects. cASTI susceptibility data were collected on 2,567 blood, urine, and respiratory isolates. Matching the data from the NHSN AR Option and the events from the cASTI data for all 3 healthcare systems resulted in 1,009 isolates. After removal of duplicate isolates, 852 matched isolates with 3,859 unique susceptibilities remained for analysis.

Of the 852 isolates, 569 (66.8%) were from healthcare system A, 168 (19.7%) were from system B, and 115 (13.5%) were from system C. Furthermore, 702 isolates (82.4%) were from urine, 97 (11.4%) were from sputum, and 53 (6.2%) were from blood. Also, 646 isolates (75.8%) were Enterobacterales (542 *Escherichia coli*, 90 *Klebsiella* spp, and 14 *Enterobacter* spp), 174 (20.4%) *Pseudomonas aeruginosa*, and 32 (3.8%) *Acinetobacter baumannii* (Table 1). Of the 852 isolates, 620 (72.8%) had susceptibility results for cefotaxime, 851 (99.9%) for ceftazidime, 678 (79.6%) for ceftriaxone, 643 (75.5%) for ertapenem, 785 (92.1%) for imipenem, and 282 (33.1%) for meropenem included in the cASTI surveillance data.

**Table 1.** Organisms and Specimen Types

Organism	No. of Isolates
*Acinetobacter baumannii*	32
Blood	6
Sputum	14
Urine	12
*Enterobacter aerogenes*	2
Urine	2
*Escherichia coli*	542
Blood	32
Sputum	19
Urine	491
*Enterobacter cloacae* complex	12
Blood	1
Sputum	2
Urine	9
*Klebsiella oxytoca*	10
Sputum	1
Urine	9
*Klebsiella pneumoniae*	80
Blood	4
Sputum	10
Urine	66
*Pseudomonas aeruginosa*	174
Blood	10
Sputum	51
Urine	113
Grand Total	852

**Fig. 2.**
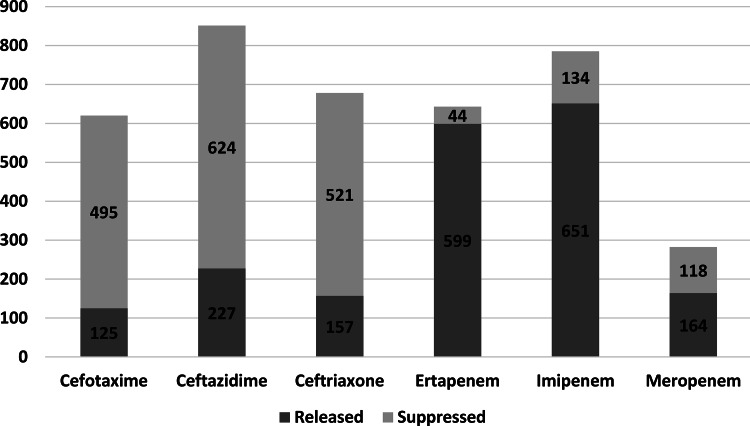
Suppressed and released results by antibiotic.

At least 1 suppressed antibiotic susceptibility result was available for 726 (85.2%) of the isolates. The greatest proportion of suppressed results was for cefotaxime (79.8%), and ertapenem had the lowest proportion of suppressed results (6.8%). The third-generation cephalosporins were all suppressed in greater proportion compared to the carbapenems (Fig. 2).

**Fig. 3.**
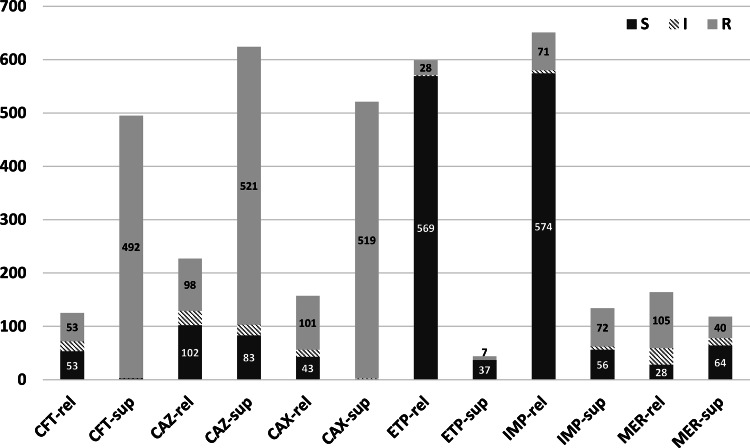
Susceptibilities for suppressed and released results for each antibiotic. Note. CFT, cefotaxime; CAZ, ceftazidime; CAX, ceftriaxone; ETP, ertapenem; IMP, imipenem; MER, meropenem; rel, released; sup, suppressed.

Of the 3,859 susceptibility results, 1,612 (41.8%) were susceptible, 140 (3.6%) were intermediate, and 2,107 (54.6%) were resistant. Figure 3 details the proportion of susceptible, intermediate, and resistant results of each antibiotic’s suppressed and released results. When compared to the cASTI data, 1,936 (50.2%) suppressed antibiotic susceptibility results were not reported in the NHSN AR Option. Of these suppressed results, 1,651 (85.2%) were resistant, 244 (12.6%) were susceptible, and 41 (2.1%) were intermediate. Of the remaining 1,923 results that were released, 456 (23.7%) were resistant, 1,369 (71.2%) were susceptible, and 98 (5.1%) were intermediate. We detected a statistically significant difference between the susceptibilities of the suppressed and released results (*P* < .0001).

System C had the highest proportion of isolates with at least 1 suppressed result in 111 of their 115 isolates (96.5%). System A had at least 1 suppressed result in 489 of their 569 isolates (85.9%), followed by system B with at least 1 suppressed result in 126 of their 168 isolates (75.0%). The difference in proportion of suppressed results among the 3 systems was significantly different (*P* < .001). Systems A and B had 85% of their Enterobacterales isolates with at least 1 suppressed result, whereas 97.9% of Enterobacterales isolates in system C had at least 1 suppressed result (Table 2). We detected no statistically significant difference when comparing the systems’ proportions of individually suppressed results (*P* = .06). System C had the highest proportion of suppression, with 296 (73.4%) of 403 results suppressed. This was followed by system A, with 1,467 (51.6%) suppressed results of 2,845 total results and system B, with 173 (28.3%) suppressed results of 611 total results. System C suppressed 85.8% susceptibility results for carbapenems analyzed, whereas system B released 99% of their ertapenem and imipenem results. System B performed no susceptibility testing on meropenem. System A suppressed 78% of susceptibility results for third-generation cephalosporins.

**Table 2.** Suppressed and Released Results by Organism and System

Suppressed and Released *Acinetobacter* Results by System, No. (%)							
**System B**							
	Ceftazidime	Cefotaxime	Ceftriaxone	Ertapenem	Imipenem	Meropenem	Total
Released	20 (87.0)	21 (91.3)	8 (34.7)	N/A	N/A	19 (82.6)	68 (73.9)
Suppressed	3 (13.0)	2 (8.7)	15 (65.2)	N/A	N/A	4 (17.4)	24 (26.1)
Total	23	23	23	N/A	N/A	23	92
**System C**							
	Ceftazidime	Cefotaxime	Ceftriaxone	Ertapenem	Imipenem	Meropenem	Total
Released	9 (100)	1 (11.1)	0 (0)	N/A	N/A	8 (88.9)	18 (60.0)
Suppressed	0 (0.0)	8 (88.9)	3 (100.0)	N/A	N/A	1 (11.1)	12 (40.0)
Total	9	9	3	N/A	N/A	9	30
Suppressed and Released Enterobacterales Results by System							
**System A**							
	Ceftazidime	Cefotaxime	Ceftriaxone	Ertapenem	Imipenem	Meropenem	Total
Released	81 (14.2)	80 (14.1)	81 (14.2)	568 (99.8)	568 (99.8)	NA	1378 (48.4)
Suppressed	488 (85.8)	489 (85.9)	488 (85.8)	1 (0.2)	1 (0.2)	NA	1467 (51.6)
Total	569	569	569	569	569	NA	2845
**System B**							
	Ceftazidime	Cefotaxime	Ceftriaxone	Ertapenem	Imipenem	Meropenem	Total
Released	25 (89.3)	24 (85.7)	11 (39.3)	23 (82.1)	14 (50.0)	21 (75.0)	118 (70.2)
Suppressed	3 (10.7)	4 (14.3)	17 (60.7)	5 (17.9)	14 (50.0)	7 (25.0)	50 (29.8)
Total	28	28	28	28	28	28	168
**System C**							
	Ceftazidime	Cefotaxime	Ceftriaxone	Ertapenem	Imipenem	Meropenem	Total
Released	2 (4.1)	N/A	48 (98.0)	8 (18.2)	0 (0.0)	12 (24.5)	70 (30.4)
Suppressed	47 (95.9)	N/A	1 (2.0)	36 (81.8)	39 (100.0)	37 (75.5)	160 (69.6)
Total	49	N/A	49	44	39	49	230
Suppressed and Released *Pseudomonas* Results by System							
**System B**							
	Ceftazidime	Cefotaxime	Ceftriaxone	Ertapenem	Imipenem	Meropenem	Total
Released	96 (82.1)	N/A	N/A	N/A	67 (57.3)	89 (76.1)	252 (71.8)
Suppressed	21 (17.9)	N/A	N/A	N/A	50 (42.7)	28 (23.9)	99 (28.2)
Total	117	N/A	N/A	N/A	117	117	351
**System C**							
	Ceftazidime	Cefotaxime	Ceftriaxone	Ertapenem	Imipenem	Meropenem	Total
Released	2 (3.6)	N/A	NA	0 (0.0)	2 (6.9)	15 (26.8)	19 (13.3)
Suppressed	54 (96.4)	N/A	NA	2 (100.0)	27 (93.1)	41 (73.2)	124 (86.7)
Total	56	N/A	NA	2	29	56	143

Note. N/A, not available.

## Discussion

The NHSN AR Option can be a valuable tool for hospitals and healthcare systems in assessing antibiotic resistance data. Moreover, this tool could used to create antibiograms for a wide range of geographical areas, which may be a project of interest for many state and local health departments.^3^ In our analysis of NHSN AR Option data, we observed a large number of susceptibility results that were not eventually reported into NHSN. The effect of antibiotic suppression that we describe in this article has significant implications for the ability of NHSN AR Option to accurately reflect antibiotic resistance rates. If data suppression is not accounted for by analysts, significant underreporting of susceptibility data will likely occur.

When stratified by drug and by susceptibility, the results were noteworthy. The third-generation cephalosporins were more frequently suppressed than the carbapenems. One possible explanation for this is that, with rare exceptions, third-generation cephalosporins are not used clinically to treat ESBL-producing pathogens, *Pseudomonas aeruginosa,* or *Acinetobacter baumannii*. Therefore, releasing these results to providers may be irrelevant or confusing. Notably, suppressing those data at the facility level is not the same as suppressing those data in the NHSN AR Option, and facilities are encouraged to report all tested results to the NHSN. Secondly, a large proportion of suppressed third-generation cephalosporins test results were resistant. Typically, the process of selective or cascading reporting results in the suppression of susceptible agents to ensure that more narrow therapies are preferentially used. The utility of suppressing resistant results would have less impact on clinical care but can have a significant impact on the development of antibiotic resistance rates using these data. We did observe variation among the different systems and their suppression rules, which affected these results. For example, one system engaged in ESBL surveillance routinely suppresses ceftriaxone results for all ESBL-producing pathogens. This could account, in part, for the high frequency of resistant ceftriaxone suppression observed. Comparatively, most of the carbapenem-suppressed results were susceptible. The CLSI publishes performance standards for antimicrobial susceptibility testing and recommends that any unexpected resistance always be reported, regardless of suppression rules.^5^

This analysis has several limitations. First, only a selected number of antibiotics relevant to the CRE, ESBL, *Pseudomonas aeruginosa*, and *Acinetobacter baumannii* surveillance that were also accessible to our EIP team were analyzed. Data regarding other agents used for treatment of these infections, such as fluoroquinolones, piperacillin-tazobactam, and fourth-generation cephalosporins were not readily available in the EIP surveillance data and were therefore not available for analysis. Thus, this analysis has described only part of the larger impact suppression likely has on the NHSN AR Option data. Secondly, we used a broad definition of the term “suppressed.” There are strong reasons for stewardship programs to implement susceptibility result suppression to guide antibiotic therapeutic choices at their facilities. However, alternative reasons (eg, formulary decisions) regarding why the test results of certain antibiotics were suppressed may have minimal impact on clinical care. Thirdly, we could not assess or describe where the suppression of antibiotic susceptibility result occurred. Susceptibility data suppression, as we describe it here, is contingent upon the types and vendors of LIMS, EMR, and surveillance systems that facilities use to report into the NHSN AR Option. We encourage facilities to assess whether implemented suppression rules are clinically relevant and to identify where the suppression occurs, which may range from the process of susceptibility testing, to transfer of data to the LIMS or EMRs, or to surveillance data reporting. As part of the validation steps of NHSN AR Option reporting, the CDC recommends examining whether susceptibility results are being suppressed due to selective or cascading reporting.^6^ If suppression is identified, the CDC recommends communicating with the facility’s surveillance software vendor to ensure that complete antibiotic resistance data are reported into the NHSN AR Option for surveillance purposes.

Currently, <1,000 hospitals report data into the NHSN AR Option in the United States, which limited our ability to analyze antibiotic resistance data across a wider geographic scale. However, as more facilities begin reporting, the NHSN AR Option has the potential to become a powerful tool to provide valuable surveillance data in the fight against antibiotic-resistant pathogens at both the facility level and at the population level. Our findings indicate that the susceptibility data suppression implemented for antimicrobial stewardship purposes or other reasons have significant impact on the accuracy and completeness of antibiotic resistance data reported to NHSN AR Option. Although it is important to fully understand the gaps in surveillance data, it is simultaneously important to support suppression rules that facilities use as a strategy to support antimicrobial stewardship efforts. If the NHSN AR Option is to be used to develop antibiogram-type data, it is critical that the CDC, the reporting facilities and healthcare systems, and other public health entities evaluate the effect susceptibility data suppression has before those data are used to make clinical and patient-care decisions.
